# A comprehensive perspective of autistic traits and catatonic symptoms in a patient with Fronto-Temporal Dementia and Bipolar Disorder: a case report

**DOI:** 10.1186/s12888-023-04709-9

**Published:** 2023-03-30

**Authors:** Liliana Dell’Osso, Ilaria Chiarantini, Chiara Bonelli, Gabriele Cappellato, Barbara Carpita

**Affiliations:** Department of Clinical and Experimental Medicine, University of Pisa, Via Roma 67, 56127 Pisa, Italy

**Keywords:** Autism Spectrum, Bipolar Disorder, Fronto-Temporal Dementia, Catatonia, Case Report

## Abstract

**Background:**

Fronto-Temporal Dementia (FTD) is a neurodegenerative disorder featuring frontotemporal lobe atrophy which leads to profound changes in behavior and cognition in the affected subjects. Considering that the onset of this type of dementia is typically characterized by the development of affective symptoms, differential diagnosis between FTD and Bipolar Disorder (BD) is particularly difficult. An important overlapping feature between BD and FTD is the presence of catatonic symptoms: Catatonia is extremely frequent in FTD, and, on the other hand, BD is the psychiatric disease with the highest frequency of association with catatonic states. In this framework, it should be noted that also Autism Spectrum conditions have been reported to show high rates of comorbidity and overlapping features with BD. In addition, subjects with autistic traits were reported to show an increased vulnerability towards the development of mood and anxiety disorders, as well as increase the risk of mood episodes with mixed features, suicidal thoughts and catatonic symptoms.

**Case presentation:**

We reported the case of a patient with a diagnosis of both BD and FTD who showed catatonic symptoms.

**Objectives:**

The aim of this case report is to evaluate the possible role of autistic traits in the illness trajectory of BD and FTD.

**Conclusion:**

This case confirms the presence of a continuum between psychiatric and neurological conditions, which should be considered as expressions of a same neurobiological system and further investigated in light of an integrative model.

## Introduction

Fronto-Temporal Dementia (FTD) is a neurodegenerative disorder characterized by frontotemporal lobe atrophy. The neuronal degeneration of these regions leads to profound changes in behavior and cognition in the affected subjects. The estimated prevalence is 15–22/100,000 subjects in the general population. Symptomatology is characterized by progressive disinhibition, apathy and loss of empathy, persevering behavior with stereotypes and functional impairment [1]. According to the literature in the field, differential diagnosis between FTD and Bipolar Disorder (BD) is particularly difficult, considering that the onset of this type of dementia is typically characterized by the development of affective symptoms. On the other hand, age-related alterations of the Central Nervous System (CNS) have been associated with the presence of BD [2]. The average age of onset for FTD is set between 45 and 65 years, although rare cases have been reported in younger people. Prodromal psychiatric symptoms are often represented by hypochondriac rumination [3]. However, the clinical presentation may vary greatly depending on the subject. Patients can experience a spectrum of symptoms ranging from disinhibition and hyperactivity to inertia and apathy, before the progressive worsening of the disease. Another overlapping feature between BD and FTD is the presence of catatonic symptoms: Catatonia is extremely frequent in FTD, and, on the other hand, BD is the psychiatric disease with the highest frequency of association with catatonic states [4, 5].

In this framework, it should be noted that also Autism Spectrum conditions have been reported to show high rates of comorbidity and overlapping features with BD [6–8]. Autism Spectrum Disorder (ASD) is a neurodevelopmental condition featuring significant difficulties in social communication and interactions, as well as restricted interests and repetitive behaviors, all of which have a substantial influence on overall functioning [9]. Patients with ASD may show different degrees of disability, including presence or not of intellectual impairment or altered language development. During the last decades, more emphasis has been placed on a dimensional approach to the Autism Spectrum, stressing the importance of investigating the presence of milder or even subthreshold forms of ASD in both children and adults [10, 11]. The interest in subthreshold forms of ASD lies in the fact that both full-threshold and sub-threshold autistic traits are considered vulnerability factors for developing other psychiatric disorders, ranging from mood and anxiety conditions to feeding and eating disorders. In this framework, DSM-5's ASD presentations could be considered as the edge of an iceberg that incorporates a wide range of potential clinical and non-clinical phenotypes. Globally, the autism spectrum may be conceptualized as a dimensional structure, or even a trans-nosographic dimension that may be the core of non-psychopathological personality features as well as the starting point for several psychopathological trajectories. These multiple pathways may be influenced by the localization, severity, and interconnections of the neurodevelopmental alteration with other biological factors as wells with environmental conditions and life events. The high rate of individual differences suggests the presence of latent dimensions rather than categories also in this scenario [12–22]. In particular, subjects with Autism Spectrum conditions, both full-threshold and subthreshold due to their increased vulnerability and their proneness to rumination, may develop stress-related symptoms or also full-blown Post-Traumatic Stress Disorder (PTSD) not only after the typical traumatic experiences described in the Diagnostic and Statistical Manual of Mental Disorder, Fifth Edition, Text Revised (DSM-5 TR) criteria for PTSD, but also after stressful life events of milder intensity, according to the complex-PTSD model [11, 17, 23–25]. More recently, several authors are stressing the possible role of subthreshold autistic traits as a possible predisposing factor for developing Catatonia and other worse clinical outcomes in patients with psychiatric disorders [26–28]. Considering the trajectory of illness among subjects with full-threshold ASD, but also that of subjects with subthreshold autistic traits, post-traumatic stress symptoms may facilitate the development of mood and anxiety disorders, as well as increase, in more vulnerable individuals, the risk of mood episodes with mixed features, suicidal thoughts and, in the most severe presentations, Catatonia [12, 13, 18, 26, 27, 29]. In this study, we aimed to discuss the case of a subject with a diagnosis of both BD and FTD who showed catatonic symptoms, evaluating the possible role of autistic traits in the illness trajectory.

## Patient information section

Mrs X.Y. is a 70 years old, married woman who lives with her husband, without a family history of mental disorders. Since adolescence, the patient was characterized by a cyclothymic-irritable temperament, with swings in mood, energy and irritability. In addition, the patient showed since childhood behavioral pattern characterized by difficulties in socio-emotional reciprocity, empathy alterations, strong adherence to routine without flexibility, narrow and repetitive interests, described as abnormal for their intensity, ascribable to a subthreshold Autism Spectrum without an evident impairment in global functioning, showing instead hyper-functional features in specific fields. As a teenager she excelled in humanities, to the study of which she devoted many hours of the day until she reached very high school performances. She was the first of her class and this stubbornness allowed her to achieve and maintain managerial roles, with a successful academic and work career. She was also very selective in her relations with a reduced ability to share interests. Described as a ruminative person, she was incapable of coping with stressful or mildly traumatic social situations, often as a result of her difficulties in social interactions and in understanding the typical signs of verbal and non-verbal communication. The patient also reported an increased sensitivity to caffeine and intolerance to stress, increased workload or to sudden changes in daily routine. The onset of clinical symptoms was at the age of 20, when the patient developed mood oscillations of both polarities, with depressive episodes, characterized by low energies and clinophilia lasting a few weeks as well as with episodes of mood elevation, increase in energy levels and activities, reduced need for sleep in association with irritability, verbal aggressive behavior towards other people (particularly family members) and subsyndromal panic attacks with cardiorespiratory symptoms (tachycardia, dyspnea). However, the patient did not seek medical help for these symptoms and the clinical picture resolved spontaneously. Subsequently, for several decades, Mrs. X.Y. reported subjective well-being, with satisfying levels of adjustment and global functioning, despite the presence of subsyndromal mood oscillations, tendency to irritability and prodigality and anxiety fluctuation. During 2012 (when the patient was 60 years old), in association with a stressful life event, the patient experienced a progressive worsening of mood, with sadness and loneliness, social anhedonia, asthenia, irritability associated with emotional lability, recriminatory thoughts towards family members, prodigality. Furthermore, her tendency to ruminative thinking related on life events, particularly social ones, typical of ASD full-threshold and subthreshold spectrum, caused frequent mood swings and alterations in her circadian rhythms, determining subtotal insomnia. This condition progressively worsened, with the patient showing increased social withdrawal, dysphoria, verbal and physical aggressive behaviors, with a further detrimental effect on her socio-emotional functioning, anxiety elevation with cardiorespiratory and neurovegetative symptoms, leading to a reduced global functioning. However, also in this case Mrs. X.Y., firmly convinced of being affected by a cancer, did not listen to family members and did not refer to mental health professionals, undergoing instead several somatic examinations such as repeated blood tests, echocardiography, cardiovascular consultations, abdominal computed tomography, endoscopy, all of which reported negative results. In 2014 (62 years), after a period of reduced symptom severity and better adjustment, the patient progressively developed aphasia and as a consequence she was examined by a neurologist, undergoing a brain Magnetic Resonance Imaging (MRI) and Positron Emission Tomography (PET). According to neuroimaging examination, cortical metabolic changes were observable especially in the frontal and left temporal areas, presumably related to a neurodegenerative condition. Mrs. X.Y., was diagnosed with Primary Progressive Aphasia and a FTD (Semantic Variant). In April 2015 (63 years), the patient developed a new episode of the mood disorder, characterized by dysphoric mood, frequent crying, high anxiety levels, motor restlessness, aggressive behaviors and prodigality, with frequent pantoclastic episodes. Subsequently the patient was examined for the first time at a Psychiatric Clinic, where she was admitted and then discharged with a diagnosis of "BD, mixed episode, in patient with FTD, type II diabetes mellitus and essential hypertension". The pharmacological therapy featured mood stabilizers (Valproic Acid 500 mg/day), antipsychotics (Perphenazine 6 mg/day), antidepressants (Paroxetine 10 mg/day) and benzodiazepines (Lorazepam 2 mg/day), with reported clinical benefit. During the following months, the patient enjoyed a partial global functioning, despite the presence of anxiety and irritability fluctuation with episodes of verbal aggressiveness. However, from 2015 to 2018 the patient experienced other three episodes of mood alteration similar to the one reported at 63 years, in particular during spring (March–April), and a progressive worsening of the global conditions, with the development of chronic impairment in linguistic expression, increasing aggressive behaviors, apathy, abulia and hypochondriac thoughts, leading to a loss of autonomy in common daily activities and personal care, requiring continuous daily assistance. In August 2018, she was admitted to a Neurology Clinic. The diagnosis of Fronto- Temporal Cognitive Decay (semantic variant type) was confirmed. In April 2021 (69 years), the patient was admitted for a second time in a Psychiatric Clinic, where she was discharged with the diagnosis of "Bipolar Affective Syndrome, maniacal episode, moderate; aphasia" and a psychopharmacological therapy based on mood stabilizers (Lithium Carbonate 300 mg/day), antipsychotics (Clozapina 75 mg/day), antidepressants (Paroxetine 20 mg/day, Sertraline 25 mg/day) with partial clinical benefit. However, due to the remaining psychopathological symptoms, associated with severe aphasia and the progressive reduction of the overall functioning, the patient was no longer self-sufficient in her daily routine and needed constant support from her family members. In February 2022 (70 years) the caregivers seek the help of another psychiatrist who changed the psychopharmacological therapy based on mood stabilizers (Lithium Carbonate 300 mg/day, Valproic Acid 250 mg/day), antipsychotics (Clozapine 100 mg/day) and antidepressants (Paroxetine 5 mg/day). Subsequently, the patient was addressed to our clinic, where she was admitted on 4 March 2022 for the treatment and investigation of the case. At a first physical examination the patient presented catatonic symptoms, including waxy flexibility, grimacing, mutism, negativism, echolalia. The patient spoke only on few occasions, with a very poor language characterized by pass-par-tout words. Furthermore, she showed an oppositional behavior, refusing food and hydration and thus requiring enteral nutrition through nasogastric intubation, temporarily replaced with parenteral nutrition for a suspected aspiration pneumonia. She was not oriented in place, time and person, and scarcely responsive to verbal and physical stimuli. In agreement with the relatives, she was transferred to Intermediate Care and subsequently in a long-term structure.

## Discussion

We described the case of a patient with a diagnosis of BD and FTD. Since the beginning of her psychopathological history, the patient reported significant subthreshold autistic traits, although apparently not affecting her global functioning. Despite the onset of the mood disorder in younger age, the patient maintained a good adjustment without pharmacological therapy for a long time, until the age of 60 years, when she experienced new episodes of the mood disorder with progressively increasing severity and associated with the onset of FTD, and, more recently, catatonic symptoms. According to the literature, subthreshold and full-threshold autistic features could be present in up to 50% of BD patients [7, 30–33]. A previous study from our group reported that BD patients with subthreshold autistic traits may show an early onset of the BD, longer hospital stay, more severe depressive symptoms, alteration of rhythmicity and suicidality thoughts across lifetime [7]. The above-described case may be in line with the literature that stressed a possible neurodevelopmental pathway underlying mood disorder: according to this hypothesis, autistic traits could be a vulnerability factor for the development of mood symptoms, especially after a history of stressful life events. The subthreshold autistic symptoms of the patient, despite their apparent non-clinical significance, might have been a ground of vulnerability for BD development, further stressing the need of early detection and prevention strategies targeting subjects with subthreshold autistic traits [12, 14, 23]. The case of Mrs. X.Y. also adds further insights on the relationship between BD and FTD. BD is the psychiatric disorder most frequently associated with dementia, while, among other kinds of dementias, FTD is often preceded by mood symptoms [34, 35]. Previous reports highlighted that manic behaviors may be an early sign of FTD, while manic or hypomanic symptoms may actually show several overlaps with those of FTD [36–38]. In addition, late-onset BD may constitute a precursor of FTD or, conversely, may be associated with cognitive and behavioral alterations similar to those reported in FTD [34, 35, 39]. This close association led an increasing number of authors to hypothesize the presence of shared vulnerability factors and neurobiological bases between FTD and BD, highlighting a continuum between psychiatric and neurological disorders [35, 39]. Despite that, research in this field is still scant [39]. Our case further supports the presence of interlaced relationships between these two conditions. While the clinical history of Mrs. X.Y. featured an earlier onset of the BD, the disorder remained of milder intensity, without requiring pharmacological treatment for several years. The severity of the mood symptoms increased only later in life, and soon after she was diagnosed with FTD. On the basis of this data, the development of FTD might be considered as a neuro-degenerative outcome of the BD. On the other hand, it is also possible that the BD symptoms would have been instead a prodromal condition of an underlying FTD. From an integrative perspective, FTD and BD could be even conceptualized as deeply interlaced conditions, sharing similar pathogenetic underpinnings, while featuring different presentations and impact on global functioning depending on the severity of the neurobiological alteration [35]. The hypothesis of a continuum between psychiatric and neurological conditions would be in line, and further expand, the increasing literature highlighting a possible neurodevelopmental approach to psychopathology. In particular, it was hypothesized that a neurodevelopmental alteration may be at the basis of different psychiatric disorders. The specific timing, severity, and location of the alteration, interacting with other biological as well as environmental factors, may lead to different psychopathological trajectories: from the more severe forms of ASD to the different forms of psychotic, mood and anxiety disorders [12–21]. In this framework, the presence of catatonic symptoms in the patient's clinical picture may be of particular interest. Catatonia is a life-threatening condition, which may be associated with different psychiatric disorders, including BD [9]. In addition, several overlaps between catatonic and autism symptoms have been highlighted in the literature, while Autism Spectrum have been recently hypothesized to be a vulnerability factor towards the development of more severe outcomes, including catatonic states, in patients with other mental disorders [12, 13, 18, 26, 27, 29]. The clinical overlap between the two disorders has been highlighted by a recent systematic review that observed that 10.4% of individuals with ASD also have catatonia [40]. Several explanations for this clinical overlap have been proposed, including a common alteration in the GABAergic system, in neuronal networks, or in the size of cerebellar structures, as well as a potential genetic connection resulting from susceptibility regions on chromosome 15. ASD and catatonia both share a wide range of clinical traits, including mutism, echolalia, stereotyped movements, repetitive behaviors, negativism, and arousal. The tendency to overestimate subthreshold catatonia and the failure to identify catatonic symptoms once they initially emerge in patients with ASD may both be caused by this clinical overlap [26, 41, 42]. Subthreshold autism spectrum, which does not significantly impair daily functioning, was also hypothesized to increase the risk of catatonia if unrecognized, possibly in line with the illness trajectory of the case presented here. In this framework, it should be noted that, as autism spectrum, and particularly subthreshold autistic traits, seems to be continuously distributed from the clinical to the general population, catatonia may as well be related to the autism dimension even when subthreshold or not clinically evident, in the framework of an intertwined relationship between the two broader dimensions of autism and catatonia spectrums [28, 42].

## Limitations of the study

Lack of ability to generalize and no possibility to establish cause-effect relationship are the limitations of this case report.

## Conclusions

The presence of catatonic symptoms, as the extreme end of the illness trajectory, in patient with autistic traits and a BD diagnosis, may further support the link between Autism and Catatonia Spectrums. On the other hand, the association between frontal lobe dysfunction and Catatonia may be considered in line with the role of frontal lobes in attentive behavior, motor and emotional functions [36]. The intertwined presentation of apparently different clinical conditions described in this case further stresses the presence of a continuum between psychiatric and neurological conditions, which should be considered as expressions of a same neurobiological system and further investigated in light of an integrative model [36].

## Data Availability

All data generated or analyzed during this study are included in this published article.

## Abbreviations

ASD: Autism Spectrum Disorder
BP: Bipolar Disease
CNS: Central Nervous System
DSM-5 TR: Diagnostic and Statistical Manual of Mental Disorder, Fifth Edition Text Revised
FTD: Fronto-Temporal Dementia
PTSD: Post-Traumatic Stress Disorder
MRI: Magnetic Resonance Imagine
PET: Positron Emission Tomography

## References

[1] Maia da Silva MN, Porto FHG, Lopes PMG, Sodré de Castro Prado C, Frota NAF, Alves CHL, Alves GS. Frontotemporal Dementia and Late-Onset Bipolar Disorder: The Many Directions of a Busy Road. Front Psychiatry. 2021; 12: 768722. 10.3389/fpsyt.2021.768722. 10.3389/fpsyt.2021.768722 PMC8674641 34925096

[2] Alves GS, Knöchel C, Paulitsch MA, Reinke B, Carvalho AF, Feddern R, Prvulovic D, Sudo FK, Pantel J, Reif A, Oertel V (2018). White Matter Microstructural Changes and Episodic Memory Disturbances in Late-Onset Bipolar Disorder. Front Psychiatry.

[3] Neary D (1996). Fronto-temporal Dementia: Nosology, Neuropsychology, and Neuropathology. Brain Cogn.

[4] Pompanin, S., Pigato, G., Roiter, B., Bussè, C., Cecchin, D., & Cagnin, A. Catatonia as Presenting Manifestation of Behavioral Frontotemporal Dementia: Insight From a PET/MRI Study. Prim Care Companion CNS Disord. 2021;23(2):20l02754. 10.4088/PCC.20l02754. 10.4088/PCC.20l02754 34000159

[5] Ducharme S, Albaugh MD, Nguyen TV, Hudziak JJ, Mateos-Pérez JM, Labbe A, Evans AC, Karama S; Brain Development Cooperative Group. Trajectories of cortical surface area and cortical volume maturation in normal brain development. Data Brief. 2015; 5: 929–38. 10.1016/j.dib.2015.10.044. 10.1016/j.dib.2015.10.044 PMC4669480 26702424

[6] Ghaziuddin M, Ghaziuddin N (2021). Bipolar Disorder and Psychosis in Autism. Psychiatr Clin North Am.

[7] Dell'Osso L., Carpita B., Bertelloni C.A., Diadema E, Barberi F.M., Gesi C., Carmassi C. Subthreshold autism spectrum in bipolar disorder: Prevalence and clinical correlates. Psychiatry Reasearc. 2019; 281: 112605. doi: 10.1016/j.psychres.2019.112605. 10.1016/j.psychres.2019.112605 31629303

[8] Munesue T, Ono Y, Mutoh K, Shimoda K, Nakatani H, Kikuchi M (2008). High prevalence of bipolar disorder comorbidity in adolescents and young adults with high-functioning autism spectrum disorder: A preliminary study of 44 outpatients. J Affect Disord.

[9] American Psychiatric Association (2022). Diagnostic and statistical manual of mental disorders (5^th^ ed., text rev.)

[10] Stewart GR, Corbett A, Ballard C, Creese B, Aarsland D, Hampshire A, Charlton RA, Happé F. Traumatic life experiences and post-traumatic stress symptoms in middle-aged and older adults with and without autistic traits. Int J Geriatr Psychiatry. 2022;37(2). doi: 10.1002/gps.5669. 10.1002/gps.5669 34994472

[11] Dell'Osso L, Carmassi C, Cremone IM, Muti D, Salerni A, Barberi FM, Massimetti E, Gesi C, Politi P, Aguglia E, Maj M, Carpita B (2022). Defining the Optimal Threshold Scores for Adult Autism Subthreshold Spectrum (AdAS Spectrum) in Clinical and General Population. Clin Pract Epidemiol Mental Health.

[12] Dell’Osso L, Lorenzi P, Carpita B (2019). Autistic traits and illness trajectories. Clin Pract Epidemiol Ment Health.

[13] Dell'Osso L, Carpita B, Muti D, Morelli V, Salarpi G, Salerni A, Scotto J, Massimetti G, Gesi C, Ballerio M, Signorelli MS, Luciano M, Politi P, Aguglia E, Carmassi C, Maj M (2019). Mood symptoms and suicidality across the autism spectrum. Compr Psychiatry.

[14] Carpita B, Cremone IM, Amatori G, Cappelli A, Salerni A, Massimetti G, Borgioli D, Carmassi C, Massai R, Dell'Osso L. Investigating the relationship between orthorexia nervosa and autistic traits ina university population. CNS Spectr. 2021; 1–8. 10.1017/S1092852921000420. 10.1017/S1092852921000420 33866990

[15] Dell'Osso L, Carpita B, Gesi C, Cremone IM, Corsi M, Massimetti E, Muti D, Calderani E, Castellini G, Luciano M, Ricca V, Carmassi C, Maj M (2018). Subthreshold autism spectrum disorder in patients with eating disorders. Compr Psychiatry.

[16] Dell'Osso L, Carpita B, Cremone IM, Mucci F, Salerni A, Marazziti D, Carmassi C, Gesi C (2020). Subthreshold Autism Spectrum in a Patient with Anorexia Nervosa and Behçet's Syndrome. Case Rep Psychiatry.

[17] Dell'Osso L, Cremone IM, Carpita B, Fagiolini A, Massimetti G, Bossini L, Vita A, Barlati S, Carmassi C, Gesi C (2018). Correlates of autistic traits among patients with borderline personality disorder. Compr Psychiatry.

[18] Dell'Osso L, Cremone IM, Amatori G, Cappelli A, Cuomo A, Barlati S, Massimetti G, Vita A, Fagiolini A, Carmassi C, Carpita B (2021). Investigating the Relationship between Autistic Traits, Ruminative Thinking, and Suicidality in a Clinical Sample of Subjects with Bipolar Disorder and Borderline Personality Disorder. Brain Sci.

[19] Carpita B, Muti D, Cremone IM, Fagiolini A, Dell'Osso L (2022). Eating disorders and autism spectrum: links and risks. CNS Spectr.

[20] Dell'Osso L, Abelli M, Carpita B, Pini S, Castellini G, Carmassi C, Ricca V (2016). Historical evolution of the concept of anorexia nervosa and relationships with orthorexia nervosa, autism, and obsessive-compulsive spectrum. Neuropsychiatr Dis Treat.

[21] Dell’Osso L, Cremone IM, Chiarantini I, Arone A, Casagrande D, Massimetti G, Carmassi C and Carpita B. Investigating Orthorexia Nervosa With the ORTO-R in a Sample of University Students with or Without Subthreshold Autism Spectrum: Focus on Dietary Habits and Gender Differences. Front. Psychiatry. 2022 July; 13: 900880. doi: 10.3389/fpsyt.2022.900880. 10.3389/fpsyt.2022.900880 PMC9330128 35911227

[22] Haslam N, McGrath MJ, Viechtbauer W, Kuppens P (2020). Dimensions over categories: a meta-analysis of taxometric research. Psychol Med.

[23] Carpita B, Muti D, Muscarella A, Dell'Oste V, Diadema E, Massimetti G, Signorelli MS, Fusar Poli L, Gesi C, Aguglia E, Politi P, Carmassi C, Dell'Osso L (2019). Sex Differences in the Relationship between PTSD Spectrum Symptoms and Autistic Traits in a Sample of University Students. Clin Pract Epidemiol Ment Health.

[24] Dell'Osso L, Carpita B, Cremone IM, Muti D, Diadema E, Barberi FM, Massimetti G, Brondino N, Petrosino B, Politi P, Aguglia E, Lorenzi P, Carmassi C, Gesi C (2019). The mediating effect of trauma and stressor related symptoms and ruminations on the relationship between autistic traits and mood spectrum. Psychiatry Res.

[25] Dell'Osso L, Muti D, Lorenzi P, Della Vecchia A, Carmassi C, Carpita B (2020). Autistic traits and rumination as vulnerability factors towards post-traumatic stress symptoms: Shaping psychopathological trajectories. J Psychopathol.

[26] Dell'Osso L, Toschi D, Amatori G, Gesi C (2022). Rethinking Catatonia: New Insights from the Autism Spectrum. CNS Neurol Disord Drug Targets.

[27] Dell'Osso L, Amatori G, Cappelli A, Cremone IM, Massimetti G, Gravina D, Nardi B, Benedetti F, Chiarantini I, Luciano M, Berardelli I, Brondino N, De Gregorio M, Deste G, Nola M, Reitano A, Muscatello MRA, Pompili M, Politi P, Vita A, Carmassi C, Maj M. Catatonia Spectrum: Validation of a Questionnaire Investigating Catatonia Spectrum. Front Psychiatry. 2022; 13:913286. doi: 10.3389/fpsyt.2022.913286. 10.3389/fpsyt.2022.913286 PMC9133529 35633780

[28] Dell'Osso L, Amatori G, Gesi C, Carmassi C (2021). A case of catatonia in the aftermath of the COVID-19 pandemic: does autism spectrum matter?. Ann Gen Psychiatry.

[29] Carmassi C, Bertelloni CA, Salarpi G, Diadema E, Avella MT, Dell'Oste V, Dell'Osso L (2019). Is There a Major Role for Undetected Autism Spectrum Disorder with Childhood Trauma in a Patient with a Diagnosis of Bipolar Disorder, Self-Injuring, and Multiple Comorbidities?. Case Rep Psychiatry.

[30] Matsuo J, Kamio Y, Takahashi H, Ota M, Teraishi T, Hori H, Nagashima A, Takei R, Higuchi T, Motohashi N, Kunugi H. Autistic-like traits in adult patients with mood disorders and schizophrenia. PLoS One 2015; 10(4): e0122711. 10.1371/journal.pone.0122711. 10.1371/journal.pone.0122711 PMC4383414 25838109

[31] Abu-Akel AM, Apperly IA, Wood SJ, Hansen PC (2017). Autism and psychosis expressions diametrically modulate the right temporoparietal junction. Soc Neurosci.

[32] Taylor MJ, Ronald A, Martin J, Lundström S, Hosang GM, Lichtenstein P. Examining the association between childhood autistic traits and adolescent hypomania: a longitudinal twin study. Psychol Med. 2021 1–10. 10.1017/S0033291721000374. 10.1017/S0033291721000374 33827724

[33] Meesters PD, Schouws S, Stek M, de Haan L, Smit J, Eikelenboom P, Beekman A, Comijs H (2013). Cognitive impairment in late life schizophrenia and bipolar I disorder. Int J Geriatr Psychiatry.

[34] Mendez MF, Parand L, Akhlaghipour G (2020). Bipolar Disorder Among Patients Diagnosed with Frontotemporal Dementia. J Neuropsychiatry Clin Neurosci.

[35] Papazacharias, Apostolos et al. ‘Bipolar Disorder and Frontotemporal Dementia: An Intriguing Association’. J Alzheimers Dis. 2017; 55(3):973–979. doi: 10.3233/JAD-160860. PMID: 27802240. 10.3233/JAD-160860 27802240

[36] Blumer D (1997). Catatonia and the neuroleptics: psychobiologic significance of remote and recent findings. Compr Psychiatry.

[37] Merikangas KR, Akiskal HS, Angst J, Greenberg PE, Hirschfeld RM, Petukhova M, Kessler RC (2007). Lifetime and 12-month prevalence of bipolar spectrum disorder in the National Comorbidity Survey replication. Arch Gen Psychiatry.

[38] Doughty CJ, Wells JE, Joyce PR, Olds RJ, Walsh AE (2004). Bipolar-panic disorder comorbidity within bipolar disorder families: a study of siblings. Bipolar Disord.

[39] Papazacharias A (2017). Bipolar Disorder and Frontotemporal Dementia: an intriguing association. J Alzheimers Dis.

[40] Vaquerizo-Serrano J, Salazar De Pablo G, Singh J, Santosh P. Catatonia in autism spectrum disorders: a systematic review and meta-analysis. Eur Psychiatry. 2021;65(1):e4. doi:10.1192/j.eurpsy.2021.2259. 10.1192/j.eurpsy.2021.2259 PMC8792870 34906264

[41] Dell’Osso L, Gesi C, Massimetti E, Cremone IM, Barbuti M, Maccariello G (2017). Adult Autism Subthreshold Spectrum (AdAS Spectrum): validation of a questionnaire investigating subthreshold autism spectrum. Compr Psychiatry.

[42] Dell'Osso L, Amatori G, Massimetti G, et al. Investigating the relationship between autistic traits and symptoms and Catatonia Spectrum. Eur Psychiatry. 2022;65(1):e81. Published 2022 Nov 4. doi:10.1192/j.eurpsy.2022.2334 10.1192/j.eurpsy.2022.2334 PMC9724219 36328964

